# Molecular basis for a new bovine model of Niemann-Pick type C disease

**DOI:** 10.1371/journal.pone.0238697

**Published:** 2020-09-24

**Authors:** Shernae A. Woolley, Emily R. Tsimnadis, Cor Lenghaus, Peter J. Healy, Keith Walker, Andrew Morton, Mehar S. Khatkar, Annette Elliott, Ecem Kaya, Clarisse Hoerner, David A. Priestman, Dawn Shepherd, Frances M. Platt, Ben T. Porebski, Cali E. Willet, Brendon A. O’Rourke, Imke Tammen

**Affiliations:** 1 Faculty of Science, Sydney School of Veterinary Science, The University of Sydney, Camden, NSW, Australia; 2 Ararat, VIC, Australia; 3 Harolds Cross, NSW, Australia; 4 NSW Department of Primary Industries, Elizabeth Macarthur Agricultural Institute, Menangle, NSW, Australia; 5 Wagga Wagga, NSW, Australia; 6 Department of Pharmacology, University of Oxford, Oxford, United Kingdom; 7 Medical Research Council Laboratory of Molecular Biology, Cambridge Biomedical Campus, Cambridge, United Kingdom; 8 The University of Sydney, Sydney Informatics Hub Core Research Facilities, Darlington, NSW, Australia; International Centre for Genetic Engineering and Biotechnology, ITALY

## Abstract

Niemann-Pick type C disease is a lysosomal storage disease affecting primarily the nervous system that results in premature death. Here we present the first report and investigation of Niemann-Pick type C disease in Australian Angus/Angus-cross calves. After a preliminary diagnosis of Niemann-Pick type C, samples from two affected calves and two obligate carriers were analysed using single nucleotide polymorphism genotyping and homozygosity mapping, and *NPC1* was considered as a positional candidate gene. A likely causal missense variant on chromosome 24 in the *NPC1* gene (NM_174758.2:c.2969C>G) was identified by Sanger sequencing of cDNA. SIFT analysis, protein alignment and protein modelling predicted the variant to be deleterious to protein function. Segregation of the variant with disease was confirmed in two additional affected calves and two obligate carrier dams. Genotyping of 403 animals from the original herd identified an estimated allele frequency of 3.5%. The Niemann-Pick type C phenotype was additionally confirmed via biochemical analysis of Lysotracker Green, cholesterol, sphingosine and glycosphingolipids in fibroblast cell cultures originating from two affected calves. The identification of a novel missense variant for Niemann-Pick type C disease in Angus/Angus-cross cattle will enable improved breeding and management of this disease in at-risk populations. The results from this study offer a unique opportunity to further the knowledge of human Niemann-Pick type C disease through the potential availability of a bovine model of disease.

## Introduction

Lysosomal storage diseases are a heterogeneous group of at least 70 disorders that stem from the dysfunctional transport and accumulation of substrates and lipids, resulting in compromised lysosomal function [1, 2]. Lysosomal storage diseases often result in premature death and efficient therapeutic interventions for most of these disorders are still being investigated [3]. Niemann-Pick disease is a group of rare neurodegenerative lysosomal storage diseases with a recessive mode of inheritance that are fatal due to impaired un-esterified cholesterol and sphingomyelin transport and metabolism [4, 5].

Niemann-Pick disease in humans is categorised into types A, B, C, and D, based on the type of lipid deficiency and clinical signs [6–9]. Causal variants for Niemann-Pick types A and B have been identified in the sphingomyelin phosphodiesterase 1 (*SMPD1*) gene, with variants in this gene resulting in decreased activity of the lysosomal enzyme sphingomyelinase [4, 10]. Causal variants for Niemann-Pick type C (NPC) have been identified within the NPC intracellular cholesterol transporter 1 (*NPC1*) gene and the NPC intracellular cholesterol transporter 2 (*NPC2*) gene, whereas the causal variant for the Novia-Scotian Niemann-Pick type D disease has been identified only in the *NPC1* gene [6, 11]. Causal variants in the *NPC1* and *NPC2* genes result in the accumulation of un-esterified cholesterol within the late endosomes and lysosomes [12–15]. Over 95% of the human NPC causative variants identified so far occur within *NPC1* [4]. The exact role of the NPC1 protein in facilitating un-esterified cholesterol transportation to target organelles and cells has not been established [6, 16], but it is postulated that the NPC1 and NPC2 proteins interact at different stages of un-esterified cholesterol export from the lysosome to other organelles [6, 17].

The age of onset and the presentation of clinical signs of Niemann-Pick disease in humans varies between each type, which can make diagnosis difficult [18, 19]. For therapeutic approaches to be of any benefit to NPC affected patients, early diagnosis of NPC is essential and the understanding of the role of the NPC1 and NPC2 proteins in transporting un-esterified cholesterol is imperative to allow for targeted therapeutic approaches [3].

Niemann-Pick disease types have also been diagnosed in a wide range of animal species, including mice (MGI: 2685089), cats (OMIA 001795–9685, OMIA 000725–9685, OMIA 002065–9685), dogs (OMIA 001795–9615, OMIA 000725–9615), a raccoon (OMIA 001795–9654) and cattle (OMIA 001795–9913) [5, 7, 12, 20–35]. The previous report of Niemann-Pick disease in Hereford cattle was identified as Niemann-Pick type A and the underlying genetic cause remains unknown [20].

Here, we present the first report and genetic characterisation of Niemann-Pick type C disease in three Australian Angus/Angus-cross calves. The aims of this study were to confirm the diagnosis of Niemann-Pick type C disease and to identify the causative mutation to improve management of this disease within the affected cattle population.

## Materials and methods

### Ethics statement

The collection of hair samples for this study was approved by the University of Sydney Animal Ethics Committee (Project No: 2016/998). Samples of affected animals and two dams were collected as part of diagnostic procedures in 2005 before commencement of this study.

### Animals

Several cases of a progressive neurological disease were reported in a herd of Australian beef cattle between 2002 and 2005. The herd consisted originally of various beef cattle breeds, but the use of purebred Angus bulls over multiple years resulted in a predominantly Angus/Angus-cross population. The same three Angus bulls were used repetitively for at least 5 years. Onset of disease in affected animals was observed from three months of age and animals died or were euthanised at about seven months of age. Clinical signs included hind limb weakness, dysmetria, incoordination, a wide based stance, walking sideways or falling over and recumbency (S1 Video) followed by death. Head tremors were observed in at least one animal. The condition was described to be exacerbated by stress, and animals were reported to be in good body condition at onset of disease.

In 2005, three affected half-siblings (calf 1, calf 2 and calf 3) were reported to the district veterinarian who commenced an investigation with the suspicion of a genetic condition. Detailed pedigree information was not available for these paternal half-siblings, although the possibility of sire daughter matings was recorded. Despite the lack of ill thrift, α-mannosidosis was considered as a differential diagnosis. DNA testing of calf 1 for the known Angus and Galloway α-mannosidosis mutations [36, 37] was conducted. All three calves were tested in the Virology Laboratory at the NSW Department of Primary Industries Elizabeth Macarthur Agricultural Institute for antibodies to bovine viral diarrhea virus (BVDV) using an agar gel immunodiffusion assay and Akabane virus, using a Simbu virus serogroup competitive enzyme-linked immunosorbent assay, as these viruses are known to cause congenital defects in cattle. All three calves were euthanised for post mortem investigation at around 6–7 months of age.

### Histopathology

Tissue samples from calf 1 (brain, spinal cord, heart, kidney, liver, lung and spleen) and calf 2 (brain, spinal cord, pituitary gland, muscle, peripheral nerve, eye, lymph nodes, thymus, thyroid, salivary gland, sections of the alimentary tract, heart, aorta, trachea, lung, pancreas, liver, kidney, spleen, adrenal gland and synovium) were collected at necropsy and fixed in phosphate-buffered formal-saline. Tissue samples from calf 3 (brain, spinal cord, heart, liver, lymph node, small intestine, adrenal gland) were fixed using Karnovski’s fluid. Tissues were embedded in paraffin wax, cut to 5 micron thick sections and stained with haematoxylin and eosin. Brain, liver and lymph node tissues were also stained with Periodic acid–Schiff, Ziehl–Neelsen and Pearl’s Prussian blue stains. For calf 1, ultrathin sections of the brain were examined.

### Fibroblast cell culture

Subcutaneous tissue was collected from calves 2 and 3 at necropsy. The tissue was initially cut into small pieces using sterile scissors and the fragments were washed in 5 x the volume of the tissue with cell culture medium (Minimal Essential Medium with Earl’s salts; MEM, MP Biomedicals) containing antibiotics (penicillin G and streptomycin sulfate) but without serum. The fragments were then transferred to a 200 mL glass conical flask containing a sterile magnetic stirring bar and 100 mL of 0.5% trypsin solution (BDH®, VWR Analytical Chemicals) in phosphate-buffered saline (PBS) containing antibiotics. The flask was placed on a stirring platform and stirred gently for 10 minutes at 37°C. The suspension was decanted through a funnel holding a fine sterile metal sieve and was mixed with 50 mL of cold cell culture growth medium consisting of MEM and 10% foetal bovine serum (FBS, Gibco™, ThermoFisher Scientific). A second lot of 0.5% trypsin solution was added to the residual tissue fragments and was stirred for 30 minutes at 37°C. The supernatant was decanted into the flask containing the first lot of supernatant and then poured into 50 mL tubes and centrifuged at approximately 800 g for 15 minutes. The resulting cell pellets were each resuspended in 2 mL of cell culture growth medium, pooled and the volume expanded to a total of 20 mL. The culture medium and cells were then incubated in two 25 cm^2^ cell culture flasks at 37°C. The growth medium was changed every 4–5 days until the monolayers were confluent.

Cells were maintained by removing the growth medium and replacing with 10 mL of maintenance medium (MEM and 2% FBS). Cells were subcultured by removing the culture medium, briefly rinsing with 1 mL of 0.025% trypsin solution in PBS containing antibiotics, removing the excess trypsin solution, adding 1 mL of fresh trypsin solution and incubating at 37°C until the cells separated from the surface of the flask. The cells were then dispersed by gentle aspiration with a pipette and 8 mL of fresh growth medium was added to the flask. The cells were then subcultured by adding 2 mL of the re-suspended cell solution to 8 mL of growth medium and transferred to a 25 cm^2^ flask. For cryopreservation, 8 mL of MEM containing 20% FBS and 10% dimethyl sulfoxide (Ajax Finechem Laboratory Chemicals) was added to the trypsinised cell suspension. Cells in lots of 1 mL were then added to cryovials that were slowly frozen to -80°C and then transferred to liquid nitrogen. On the first occasion that cells from an individual animal were frozen, 2 mL of the cell suspension solution was added to 18 mL of growth medium and two 25 cm^2^ flasks were seeded and incubated until the viability of the frozen cells had been confirmed.

In 2005, fibroblast cell cultures were submitted to Professor John Hopwood at the Lysosomal Diseases Research Unit, Adelaide, Australia, for initial phenotype characterisation. During 2015 and 2017, RNA was extracted from fibroblast cell cultures for sequencing of cDNA, and the NPC phenotype was validated in these fibroblast cell cultures as described below.

### DNA and RNA isolation

Genomic DNA was extracted from EDTA blood samples from calf 1, calf 2 and calf 3, as well as from the two obligate carrier dams of calf 2 and calf 3 using the UltraClean® Tissue & Cells DNA Isolation Kit (Mo Bio Laboratories Inc.) following the manufacturer’s protocol. Genomic DNA was isolated from hair roots from 403 Angus/Angus-cross animals collected in 2016 and 2017 from the original herd using a standard hair digest protocol [38].

RNA was extracted from cultured fibroblast cells from calf 2 and calf 3 and one unrelated Angus animal using the RNeasy Mini Kit (QIAGEN). Extraction was performed according to the manufacturer’s protocol with the addition of 600 μL of RLT buffer to lyse cells.

DNA and RNA concentration and purity were measured using the NanoDrop 8000 spectrophotometer (ThermoFisher Scientific).

### SNP genotyping

DNA samples of affected calves 2 and 3 and their two obligate carrier dams were submitted to the Australian Genome Research Facility (AGRF) for genotyping with the GeneSeek® Genomic Profiler Bovine HD Chip 80K chip (Neogen). Runs of homozygosity (ROH) were computed using PLINK v1.07 [39]. Threshold values of 80% and 0.01 were used for single nucleotide polymorphism (SNP) call rate, and minor allele frequency ROH were identified using the–homozyg command. A minimum length for a ROH was pre-set at 5 megabases (Mb), spanning over a minimum of 100 SNPs to define a ROH. In order to account for 1% error in genotyping calls, one heterozygote and up to two missing genotypes were allowed for each ROH. The ROH were visualised using R software [40].

### RT-PCR and Sanger sequencing

**Fig 1:**
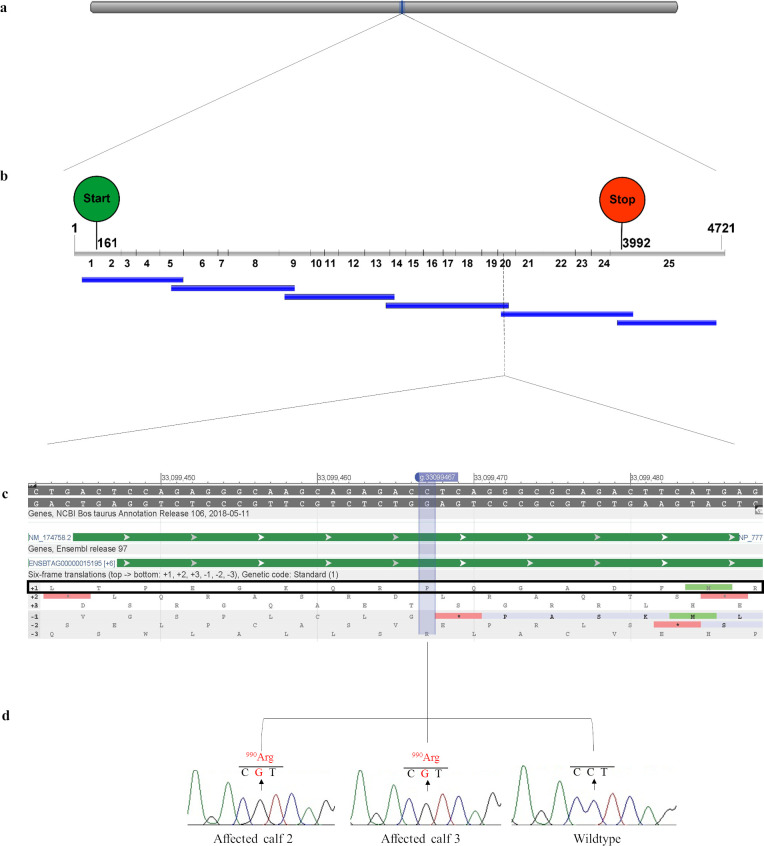
Schematic diagram of the *NPC1* gene showing the location of the candidate causal mutation NM_174758.2: g.33099467C>G with chromatograms from Sanger sequencing data for two affected calves and a wildtype control. (a) Location of the bovine *NPC1* gene, Chr24: 33058694–33105394 on the ARS-UCD1.2 bovine genome assembly. (b) Position of the six overlapping RT-PCR products (blue bars) in relation to the *NPC1* cDNA containing 25 exons (grey bars) with a transcript length of 4721 bp (ENSBTAT00000020219.5). The start codon (ATG) is indicated by the green circle and the stop codon (UAG) is indicated by the red circle, with the cDNA location of the first nucleotide of the start and stop codon given. (c) Genomic region containing the C>G missense variant with protein translation frames obtained from NCBI Genomic Data Viewer (NCBI, accessed 12^th^ August 2019, <https://www.ncbi.nlm.nih.gov/genome/gdv/browser/?context=genome&acc=GCF_002263795.1>). The reading frame is identified by a black box. (d) Sanger sequencing chromatograms for the two affected calves and a wildtype control.

PrimerBLAST [41] was used to design 6 primer pairs to amplify the cDNA (ENSBTAT00000020219.5) of the positional candidate gene *NPC1* (Table 1 and Fig 1).

**Table 1:** Primer sequences for amplification of cDNA for NPC1 and their location in the ENSBTAT00000020219.5 transcript.

Primer name	Primer sequence 5’-3’	Exon	Location	Product size (bp)
LSD_F1	CTTGGTTTCCTCCCTCCGC	1	54–72	737
LSD_R1	TGAAAAGACGGGGGTGATGG	5	771–790	
LSD_F2	GCGCCTGCAATGCTACTAAC	5	705–724	902
LSD_R2	CAGGATGGTGCAGTTCTGGT	9	1587–1606	
LSD_F3	ACAACGAGACTGTGACCCTG	9	1533–1552	799
LSD_R3	ACCTGTTGGTCGAGGGTTTC	14	2312–2331	
LSD_F4	CCTGGTCCAGACCTACCAGA	13	2272–2291	895
LSD_R4	GAACATGGGCAGGAACCTCA	20	3147–3166	
LSD_F5	CAGAGGGCAAGCAGAGACC	20	3111–3129	965
LSD_R5	GTTCAACCGAGCCTCGACA	25	4057–4075	
LSD_F6	AGGTACAGAACGAGAACAGCTC	25	3961–3982	722
LSD_R6	ACCCAGTATCCACATCTAGGAG	25	4661–4682	

Six overlapping reverse transcriptase PCRs (RT-PCR) were performed using a Mastercycler® pro and Mastercycler® (Eppendorf) in 50 μL volumes comprised of RNase-free water, 2.5 mmol/L MgCl_2_ pH 8.7, 0.8 mmol/L TrisCl pH 9.0, 4 mmol/L KCL, 0.04 mmol/L DTT, 0.004 mmol/L EDTA, 2.0 mM dNTPs, 0.6 μM of each primer (Table 1) and 20–30 ng/μL of RNA from calf 2 to amplify the entire *NPC1* cDNA. The denaturation step was performed at 95°C for 15 minutes, followed by 40 cycles consisting of a denaturation step for 1 minute at 94°C, annealing at 55°C and extension at 72°C. A final step was performed at 72°C for 10 minutes with a hold step at 15°C. For calf 3 and an unrelated Angus control the protocol was modified to amplify the region containing the NM_174758.2:c.2969C>G variant using primers LSD_F4 and LSD_R5 (Table 1). A touchdown RT-PCR was performed with a reverse transcription step at 50°C for 30 minutes, followed by denaturation at 95°C for 15 minutes. Cycling commenced at 94°C for 1 minute, followed by annealing at 65–55°C for 1 minute and extension at 72°C for 2 minutes for 11 cycles, with the annealing temperature decreasing by 1°C each cycle. A further 29 cycles were performed with denaturation at 94°C for 1 minute, annealing at 55°C for 1 minute and extension at 72°C for 2 minutes. A final extension step at 72°C for 1 minute concluded the PCR.

PCR products were purified using the MinElute PCR Purification Kit (QIAGEN) or bands were excised from a 2% agarose gel using the Wizard® SV Gel and PCR Clean-up System (Promega) before submission to AGRF for DNA sequencing. Sequencing data was analysed using Sequencher® (version 5.3, Gene Codes Corporation,) by aligning the sequences to NM_174758.2 to identify variants. Variants were compared to the variant database in Ensembl [42] and predicted impacts of novel variants on protein function were determined by SIFT analysis [43]. Cross-species NPC1 protein alignments were conducted using T-Coffee [44] and BOXSHADE (v3.2) across ten species.

### Genotyping assays

Two genotyping tests were developed for the NM_174758.2:c. 2969C>G missense variant in the *NPC1* gene to confirm segregation with disease and to estimate the allele frequency in the current herd (n = 403).

#### Genotyping by real-time PCR

Real-time PCR was performed using the ViiA™ 7 system (Applied Biosystems™) in a final reaction volume of 20 μL. Each reaction contained 1 x TaqMan® Genotyping Master Mix (Applied Biosystems™), 900 nmol/L of assay specific primers (Table 2) (Sigma-Aldrich), 10 mmol/L of allele specific probes (LGC Biosearch Technologies) (Table 2) and 15–30 ng of genomic DNA. Each PCR commenced with a pre-read stage at 60°C for 30 seconds followed by an initial denaturation at 95°C for 10 minutes. Denaturation then occurred at 95°C for 10 seconds followed by annealing/extension at 62°C for 60 seconds for 50 cycles, with a post-read stage at 60°C for 30 seconds. Genotypes were analysed using the ViiA™ 7 software version 1.1 (Applied Biosystems™).

**Table 2:** Primer and probe sequences for the real-time PCR assay and primer sequences for the RFLP PCR assay.

Primer or probe name	PCR type	Primer sequence 5’-3’	Product size (bp)
NP2016_F	Real-time PCR	GGTCAACCCTACCTGTGTC	103
NP2016_R		GTCGGAGAGGAACATGGG	
NP_wildtype		CAAGCAGAGACCTCAGGGCGCAGAC	
NP_mutant		CAAGCAGAGACGTCAGGGCGCAGAC	
NP2016_RFLP_F	PCR-RFLP	GAGGTTGTCCTTAAAGCAGGCA	331
NP2016_R		GTCGGAGAGGAACATGGG	

#### Genotyping by PCR and restriction enzyme analysis

PCR was performed using a Mastercycler® pro (Eppendorf) using primers (Table 2) in 20 μL volumes comprised of 10 mmol/L Tris-HCL pH 8.3, 50 mmol/L KCL, 1.5 mmol/L MgCl_2_, 0.1 mM dNTPs, 0.5 μM of each primer (Table 2), 0.5 U of Q solution, 0.05 U of Taq polymerase (Roche) and 15–20 ng/μL of target DNA. The initial denaturation step was performed at 94°C for 3 minutes, followed by 45 cycles consisting of a denaturation step at 94°C for 10 sec, annealing at 60°C for 15 sec and extension at 72°C for 20 sec. A final extension step was performed at 72°C for 2 minutes with a hold step at 15°C.

Restriction enzyme digestions were performed in final volumes of 15 μL containing 10 μL of PCR product and 5 μL of enzymatic solution (1 x PBS, 3.7 x 10X NEBuffer^TM^1 and 3 U of *HpyCH4*IV enzyme) (New England BioLabs Inc.) at 37°C overnight. Products were visualised on a 3.7% agarose gel or the 2100 Bioanalyzer Instrument using the 2100 Expert Software (version B.02.10.SI764) (Agilent Technologies).

### Characterisation of NPC1 phenotype in fibroblast culture

Fibroblasts from affected calves 2 and 3 and a non-related Angus control from a different study were shipped to the University of Oxford, Oxford, United Kingdom as CO_2_ equilibrated growing cultures in an insulated polystyrene box.

#### Activity assay for α-mannosidosis

The α-mannosidase activity was assayed using 4-Methylumbelliferyl-α-D-Mannopyranoside (4MU-α-Mann) as substrate, conducted in triplicate to investigate the possibility of differential diagnosis of α-mannosidosis in a wildtype control and affected calves 2 and 3. Briefly, hydrolysis of 4MU-α-Mann (Sigma-Aldrich) was conducted at 37°C in 100 mm of sodium acetate buffer (pH 4.0) using 10 μl of fibroblast homogenates at a final volume of 50 μl after addition of substrate (3.00 mm final concentration 4MU-α-Mann). Reactions were stopped by the addition of 0.5M Na2CO3. Fluorescence of released 4MU was measured using a Clariostar 96-well plate reader (Excitation 365nm and Emission 450nm).

#### Lysotracker

Bovine fibroblasts (1 × 105, in triplicate) were stained with 0.5 ml of 200 nM Lysotracker-green DND-26 (Invitrogen) in PBS for 10 minutes in the dark at room temperature. Cells were centrifuged for 5 minutes at 2000 rotations per minute, re-suspended in 0.5 ml FACS buffer (PBS+1% FCS) and kept on ice for a maximum of 1 hour prior to flow cytometric analysis (BD Biosciences FACSCanto II). The cytometer was calibrated using Cytometer Setup and Tracking beads (BD). Samples were acquired with gating on singlets (FSC-H versus FSC-A). In total, 10,000 singlet events were collected. The mean fluorescence was calculated using FACSDiva software (BD) and the mean of the triplicate reading plotted.

#### Sphingoid base measurements

Sphingoid bases (sphingosine and sphinganine) were extracted from 100 μL fibroblast homogenates in 500 μL of chloroform:methanol (1:2 v/v) followed by sonication for 10 minutes at room temperature. Subsequently, 1M 500 μL sodium chloride, 500 μL chloroform, and 3M 100 μL sodium hydroxide were added to the samples, and vortexed every 5 minutes for 15 minutes at room temperature. Homogenates were centrifuged at 13,000 g for 10 minutes, and the lower organic phase retained. Sphingoid bases were purified from the samples by pre-equilibrating SPA-NH2 columns with 2 x 1 mL chloroform followed by sample elution with 3 x 300 μl acetone. The samples were dried under nitrogen. Lipids were re-suspended in 50 μL pre-warmed 37°C ethanol and 50 μL o-phthaldialdehyde (OPA) labelling solution (1 mg OPA/20 μL ethanol/1 μL 2-mercaptoethanol; dilution 1:2000 in 3% boric acid pH 10.5) was added. Samples were kept at room temperature in the dark for 20 minutes and vortexed every 10 minutes. Samples were buffered with 100 μL methanol:5mM Tris pH 7 (9:1) and centrifuged at 5,000 g for 2 minutes. Supernatants (150 μL) were loaded onto a reverse phase HPLC. Chromatography was carried out using a mobile phase of 85% acetonitrile/15% H_2_O at a flow rate of 1.0 ml/minute. The orthophthaldehyde-labelled derivatives were monitored at an excitation wavelength of 340 nanometres (nm) and an emission wavelength of 450 nm. Quantification of trace peak area was carried out using EZChrom Elite software v3.2.1 (http://www.jascoinc.com/ezchrom).

#### Cholesterol measurements

Cholesterol was measured with the Amplex Red kit (Molecular Probes) according to the manufacturer's instructions. Briefly, cell and tissue homogenates in 100 μL milliQ H_2_O were Folch extracted and dried down under nitrogen. The pellets containing cholesterol were resuspended in 1X reaction buffer, and 50 μL was loaded for each sample in a flat bottom 96-well plate. The reaction was initiated by adding working solution per sample (25% Amplex® Red, 2 U/mL HRP stock solution, 2 U/mL cholesterol oxidase stock solution, 0.2 U/mL cholesterol esterase stock solution in 1X reaction buffer). Samples were incubated at 37°C for 30 minutes and the fluorescence was measured in a microplate reader (Optima, BMG Labtech) using excitation in the range of 530–560 nm and emission detection at ~590 nm.

#### Filipin staining

Free cellular cholesterol were visualised with filipin (from *Streptomyces filipinensis*) (Sigma-Aldrich). Cells were fixed with 4% paraformaldehyde, washed 3 x PBS and incubated with 1.5 mg/mL glycine for 10 min to quench auto-fluorescence. Cells were then incubated with filipin (0.05 mg/mL in PBS/10% FBS/0.2% Triton x100) for 2 hours, washed 3 x PBS and visualized by Leica-SP8 confocal microscope.

#### Cholera toxin B staining

GM1 staining with cholera toxin was conducted on fixed and permeabilised cells by incubating cells with 1:1000 dilution of stock solution (1 mg/ml) for 2 hours in 0.5% bovine serum albumin (BSA) in PBS washed 3 x with PBS. Cholera toxin B staining of cells were visualised by Leica-SP8 confocal microscope.

#### Image quantification

Filipin and cholera toxin signals were quantified by acquiring mean gray value for each defined cell (ROI) using Fiji Software (http://fiji.sc/Fiji) [45].

#### Normal phase HPLC for glycosphingolipids in cultured bovine fibroblasts

Glycosphingolipids (GSLs) were analysed essentially as described previously [46]. Lipids from cultured bovine fibroblasts were extracted with chloroform and methanol overnight at 4°C. The GSLs were then further purified using solid-phase C18 columns (Telos, Kinesis). After elution, the GSL fractions were dried down under a stream of nitrogen at 42°C and treated with recombinant ceramide glycanase (rEGCase I, prepared by Genscript and kindly donated by Orphazyme) to obtain oligosaccharides from more complex GSLs. The liberated free glycans were then fluorescently-labelled with anthranillic acid (2AA). To remove excess 2AA label, labelled glycans were purified using DPA-6S SPE columns (Supelco). Purified 2 2AA-labelled oligosaccharides were separated and quantified by normal-phase high-performance liquid chromatography (NP-HPLC) as previously described (Neville et al, 2004). The NP-HPLC system consisted of a Waters Alliance 2695 separations module and an in-line Waters 2475 multi λ-fluorescence detector set at E x λ360 nm and Em λ425 nm. The solid phase used was a 4.6 x 250 mm TSK gel-Amide 80 column (Anachem). A 2AA-labelled glucose homopolymer ladder (Ludger) was included to determine the glucose unit values (GUs) for the HPLC peaks. Individual GSL species were identified by their GU values and quantified by comparison of integrated peak areas with a known amount of 2AA-labelled BioQuant chitotriose standard (Ludger). Protein concentration in fibroblast homogenates was determined using the BCA assay (Sigma-Aldrich).

### Structural analysis

#### Homology modelling of bovine NPC1

As of writing, there are currently no wildtype bovine NPC1 (NP_777183.1) structures available in the Protein Data Bank (PDB) (http://www.rcsb.org/) [47]. We therefore decided to build a homology model based on a recent cryoelectron microscopy structure of the human NPC1 protein (PDB: 6UOX) [48]. This was performed using Modeller v.9.24 [49], where 100 models were constructed using the full-length wildtype bovine NPC1 sequence (NP_777183.1) and the lowest Discrete Optimized Protein Energy (DOPE) scoring model was selected for further evaluation. A model of bovine NPC1 containing the p.P990R mutation was created using the PyMOL Molecular Graphics System, v.2.3.4 (Schrödinger, LLC) and selecting the rotamer of best fit.

#### Molecular dynamics simulations

All steps of the simulation system setup were performed using the CHARMM-GUI webserver’s membrane builder tool [50, 51]. Initially, chain termini were capped with neutral groups (acetyl and methylamide). Residues were protonated as per their expected states at pH 7. Disulfide bonds were added based on homology to structures of human NPC1. As NPC1 is an endosomal trans-membrane protein, we opted to explicitly model the protein in a 100% POPC (1-palmitoyl-2-oleoyl-sn-glycero-3-phosphocholine) lipid bilayer. The PPM web server was used to determine the rotational and translational position of the transmembrane region [52]. The simulation box was set to be a rectangular system with a XYZ size of 100.12 x 100.12 x 219.21Å, which resulted in 121 and 109 lipids in the top and bottom layer respectively. Ions were added to the system to yield a NaCl concentration of 150 mM, before being solvated with TIP3P water [53]. Protein and ions were modeled with the AMBER ff14SB force field [54]. Lipids were modeled with the LIPID14 force field [55]. All bonds involving hydrogen atoms were constrained to their equilibrium lengths with the SHAKE algorithm [56]. The resulting systems were subjected to at least 5,000 steps of energy minimisation to remove any clashes, followed by an equilibration protocol with periodic boundaries. The equilibration protocol was provided by the CHARMM-GUI web server, and consists of 6 steps with harmonic positional restraints applied. Restraints started at 10 kcal/mol/Å^2^ for backbone atoms, 5 kcal/mol/Å^2^ for sidechain atoms, 10 kcal/mol/Å^2^ for ions and 2.5 kcal/mol/Å^2^ for membrane atoms, and were gradually relaxed over 6 cycles for 125 picoseconds (ps) each at a time step of 1 femtosecond (fs). Temperature was initialised using random velocities with a fixed target of 311.15 Kelvin, maintained by a Langevin thermostat. After 2 cycles of equilibration, constant pressure control was introduced using a semi-isotropic Berendsen barostat, at a target pressure of 1.0 bar. After 3 cycles of equilibration, positional restraints continued to be relaxed, and the cycle time was extended to 500 ps and the time step was increased to 2 fs. Throughout, a 9 Å cut-off radius was used for range-limited interactions with Particle Mesh Ewald electrostatics for long-range interactions. Production simulations were conducted in an isothermal-isobaric (NPT) ensemble as described above, with a 2 fs time step for 500 ns total and run in triplicate from independent starting velocities using Amber v.19.19.12 [57, 58] and PMEMD.cuda on Nvidia V100 GPUs.

Simulation trajectories were processed and analysed using a combination of AmberTools, PyMol, VMD and custom Python (v3.7) (https://www.python.org/) scripts.

## Results

### Clinical findings

Between 2002 and 2005, a small proportion of calves from a commercial beef herd in Australia presented with progressive neurological signs (S1 Video). The three affected calves (calves 1, 2 and 3) investigated in 2005 were reported to be sired by the same bull. Herd and grazing history did not support plant toxicity as a cause of disease and all three affected calves tested negative for BVDV and Akabane virus. Due to a possible history of inbreeding, a recessive inherited disease was considered.

Based on the suspicion of an inherited neurodegenerative disease, calf 1 was genotyped for two mutations known to cause bovine α-mannosidosis, a lysosomal storage disease that has been previously described in Angus and Galloway cattle (OMIA 000625–9913) [36, 37, 59]. The affected calf was homozygous wildtype for both bovine α-mannosidosis mutations.

### Pathology

**Fig 2:**
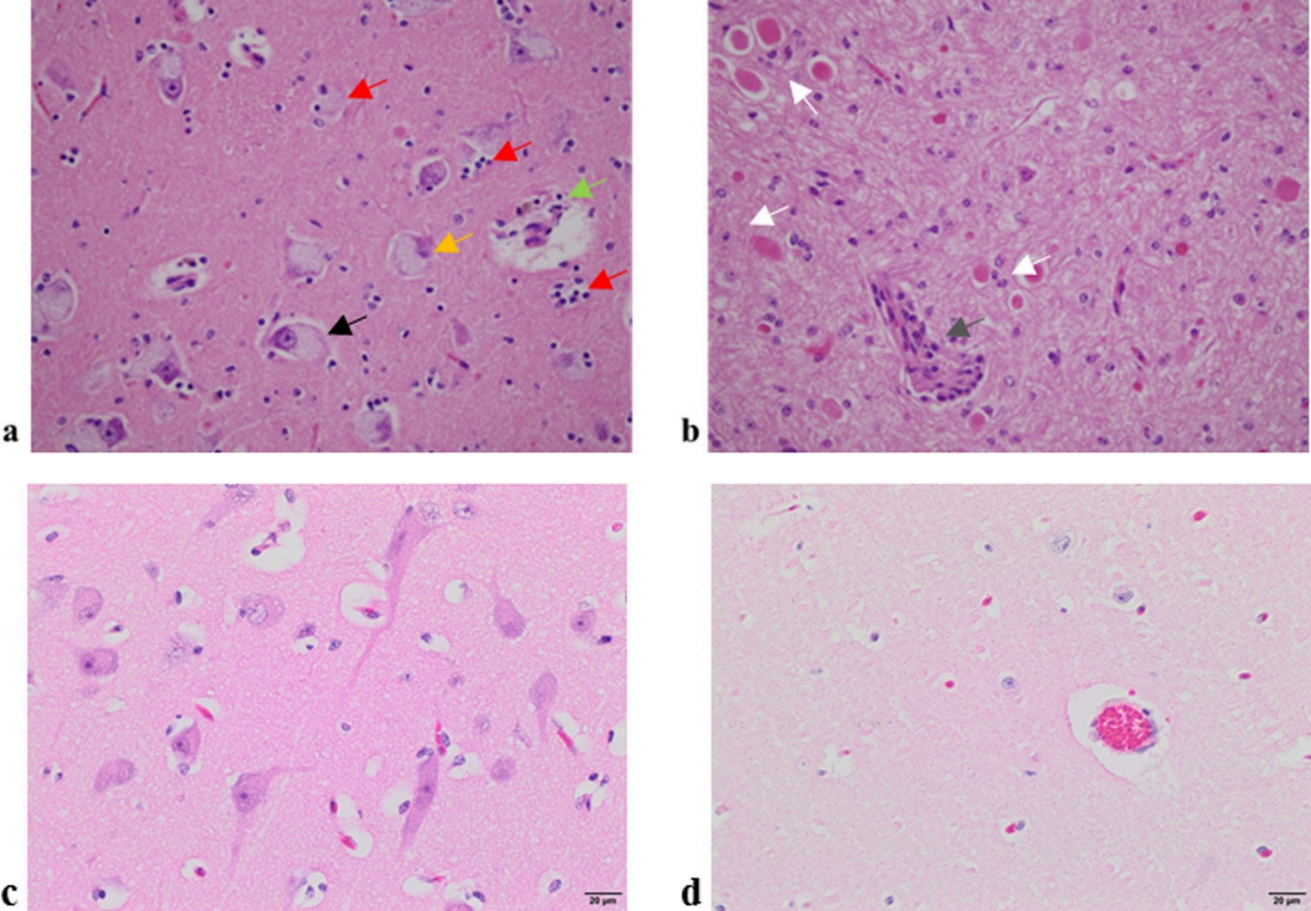
Haematoxylin and eosin staining of 5 micron thick sections from two affected and two unaffected calves at 40x magnification. (a) Thalamus of calf 2 showing nuclei with foamy, granular basophilic cytoplasm (black arrow), degenerate neurons with a condensed and basophilic nucleus (orange arrow) with the ensuing phagocytosis (red arrows) and macrophages with brown-pigmented cellular debris accumulating around a blood vessel (green arrow). (b) Cerebellar white matter of calf 3 showcasing widespread foamy vacuolation of the cytoplasm of neurons and glia, with eosinophilic and axonal swellings (white arrows) and macrophages and lymphocytes surrounding a small blood vessel (grey arrow). (c) Thalamus from a healthy calf and (d) cerebellum from a healthy calf.

At necropsy of calf 1, excessive cerebral spinal fluid was noted and no gross pathology was reported for calves 2 and 3. Histologically, brain tissue from these calves presented with similar widespread, foamy vacuolation of the cytoplasm of neurons and glia, with eosinophilic and axonal swellings (spheroids) (Fig 2).

This effect was observed throughout the brain and spinal cord with more intense degeneration in selected areas. In the cerebellum, there was marked vacuolation of Purkinje cells with prominent, swollen proximal axonal processes in the internal granular cell layer (torpedoes). There was extensive Wallerian-type degeneration of white matter tracts in the cerebellum particularly, with numerous digestion chambers containing macrophages and detritus. In calf 2, with perhaps the most advanced disease, there was marked astrocytosis and microgliosis with a large proportion of degenerated neurons and neuronophagic nodules present. The chronicity of the process was reflected in the presence of golden-brown lipid breakdown products, seen as globules in the cytoplasm of perivascular macrophages (globoid cells). In the heart there was marked hypertrophy of Purkinje cells, with vacuolation of the cytoplasm. There were large aggregates of foamy macrophages at the cortico-medullary junction in lymph nodes. Brain, liver and lymph node tissues stained with Periodic Acid–Schiff stained weakly due to soluble storage materials being present, and not polysaccharides which can be lost during tissue processing. Tissues stained with Ziehl–Neelsen were negative and Pearl’s Prussian Blue stains were negative for iron. The examination of ultrathin brain sections using electron microscopy revealed material that was finely granular, sometimes within intracytoplasmic, membranous bodies. The identification of further intracellular storage material was difficult to examine, due to the appearance of general stored cellular debris. Examination of these sections did not reveal any micro-organisms or viral particles. Based on these findings a lysosomal storage disease was diagnosed.

Analysis of fibroblasts from affected calves 2 and 3 and a normal Angus control at the Lysosomal Storage Disease Research Unit in Adelaide, South Australia in 2005, was suggestive of Niemann-Pick disease due to positive filipin staining. However, these results were inconclusive due to variability in filipin staining across cells.

### SNP genotyping and homozygosity analysis

**Fig 3:**
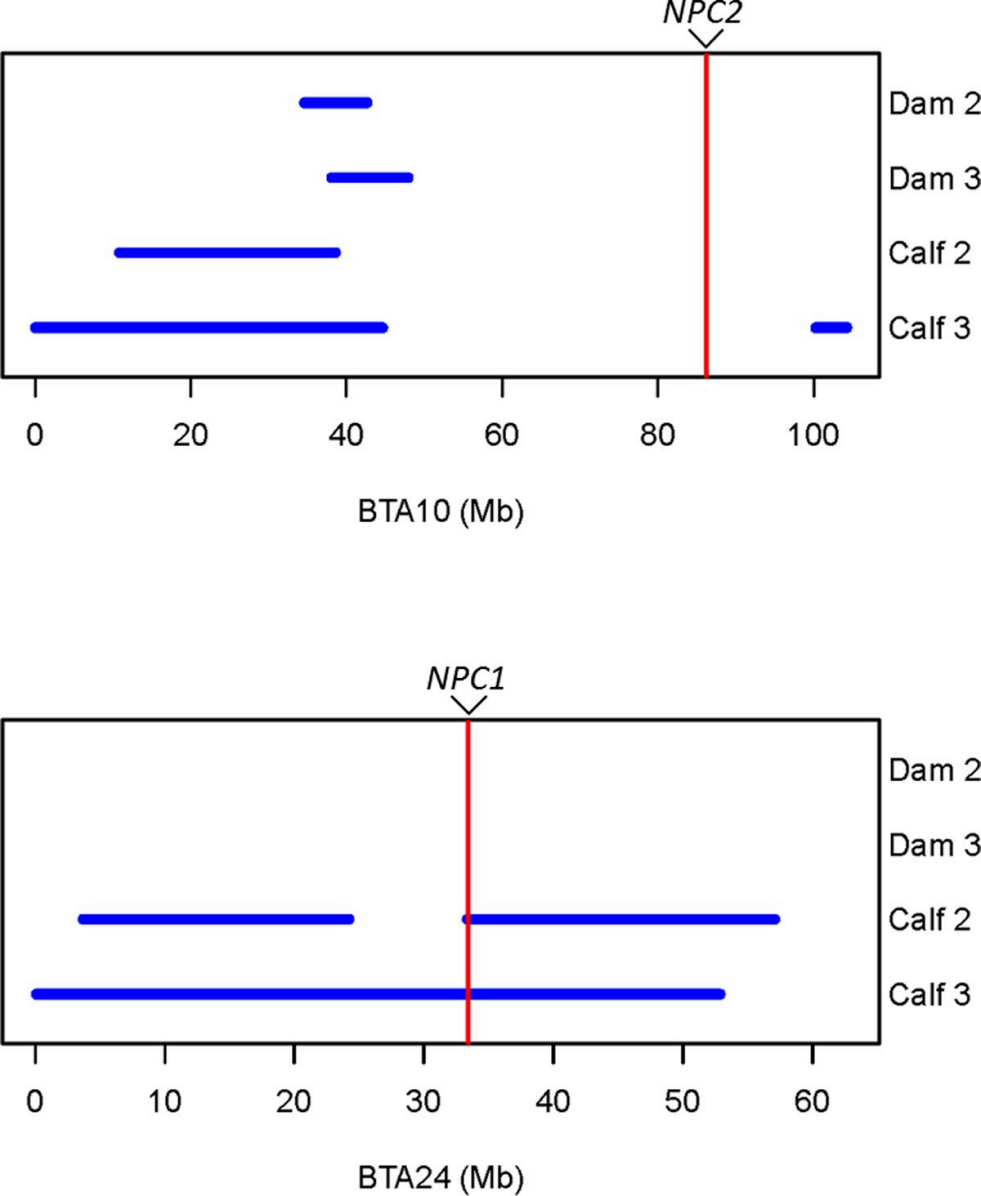
Two Regions of Homozygosity (ROH) for affected calves 2 and 3 and their respective dams showing approximate locations for two NPC candidate genes of interest based on the UMD_3.1 bovine genome assembly. (a) A shared ROH on chromosome 10 did not contain the *NPC2* gene NM_173918:g. 86,170,653–86,179,237 and (b) A shared ROH on chromosome 24 contained the *NPC1* gene NM_174758:g. 33,438,455–33,485,188.

Call rates for the SNP genotyping data for two affected and two obligate carrier animals were on average 99.81%. A sliding window of 100 SNPs identified a shared ROH between both calf 2 and calf 3 on *Bos taurus* autosome (BTA) 24 and BTA26 that were not shared with the obligate carrier dams of each calf. These ROH windows were approximately 40 Mb and 6 Mb in size respectively (Fig 3). Bovine homologs for the genes that cause Niemann-Pick disease in humans (*SMPD1*, *NPC1* and *NPC2*), are located on BTA15, BTA24 and BTA10 respectively. The *NPC1* gene was considered as a positional candidate gene based on the described gene function in humans and mice, and the phenotypes associated with a defective NPC1 protein.

### Identification of a likely causal variant

**Fig 4:**
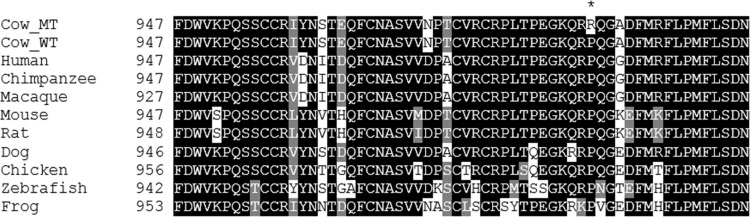
Multiple species NPC1 protein alignment using T-Coffee and BOXSHADE was completed using accessions NP_777183.1 (*Bos taurus*), NP_000262.2 (*Homo sapiens*), XP_001155285.1 (*Pan troglodytes*), XP_002800934.1 (*Macaca mulatta*), NP_032746.2 (*Mus musculus*), NP_705888.2 (*Rattus norvegicus*), NP_001003107.1 (*Canis lupus familiaris*), XP_419162.3 (*Gallus gallus*), NP_001230804.1 (*Danio rerio*) and XP_004915269.1 (*Xenopus tropicalis*). CowMT refers to the mutant *Bos taurus* sequence and CowWT refers to the wildtype *Bos taurus* sequence. The predicted change from the proline to arginine in the affected calves is highlighted by an asterix (*) in the CowMT sequence.

A likely causal homozygous missense variant (NM_174758.2:c.2969C>G) was identified in calf 2 by sequencing six overlapping RT-PCR products that spanned all 25 exons of the bovine *NPC1* gene (Table 2 and Fig 1). The homozygous c.2969C>G missense variant results in the substitution of a proline residue for arginine at position 990 of the NPC1 protein (Fig 4). To predict the effect of the p.P990R missense mutation on function, SIFT [43] and PolyPhen-2 [60] were used. SIFT calculated a score of 0, which is interpreted to be ‘damaging’ to function across all three *NPC1* transcripts. PolyPhen-2 calculated a score of 0.999, which is interpreted to be ‘probably damaging’ with a high probability. Although both SIFT and PolyPhen-2 are trained on human datasets, homology (88.6% identity) and conservation of this position with human NPC1 is high, and both methods are in agreement that this is a damaging mutation.

A further 11 variants were identified in the sequence data and included a novel heterozygous missense variant and 10 homozygous previously reported variants (Table 3). Of the previously reported variants, 9 were synonymous and one (p.F390L) was a missense variant, with a calculated SIFT score of 0.64 (tolerated) and a PolyPhen-2 score of 0.0 (benign) and is therefore unlikely to be disease-causing.

**Table 3:** Variants identified in Sanger sequencing of *NPC1* in affected calf 2.

Genomic location (ARS-UCD1.2)	Ref	Alt	Variant ID	Effect on protein (Accession ID: NP_777183.1)	SIFT	Poly Phen-2	Genotype of individual
Chr24:33058797	G	A	-	-	-	-	GA
Chr24:33076979	T	C	rs382241638	p.Arg179 =	-	-	CC
Chr24:33076997	T	C	rs380249089	p.Asn185 =	-	-	CC
Chr24:33080459	T	C	rs136708615	p.Ala333 =	-	-	CC
Chr24:33080538	T	C	rs134085739	p.Phe360Leu	0.64	0.0	CC
Chr24:33080768	T	C	rs137533522	p.Ala436 =	-	-	CC
Chr24:33084639	A	G	rs109486809	p.Gly535 =	-	-	GG
Chr24:33090972	C	T	rs133148564	p.Ile654 =	-	-	TT
Chr24:33091053	C	T	rs137071819	p.Pro681 =	-	-	TT
Chr24:33095637	T	C	rs110517880	p.Ile770 =	-	-	CC
Chr24:33098763	A	G	rs132653527	p.Ala969 =	-	-	GG
**Chr24:33099467**	**C**	**G**	**-**	**p.Pro990Arg**	**0**	**0.999**	**GG**

The likely causal variant (bold) was also Sanger sequenced in calf 3 and a wildtype control.

The likely causal variant c.2969C>G was validated using Sanger sequencing of a targeted RT-PCR product in calves 2, 3 and an unrelated control. Validation using two genotyping assays was conducted for all three affected calves, the dams of calves 2 and 3, and 403 animals from the original herd with concordant results (S1 Fig). All three affected calves were homozygous for the c.2969C>G variant, and both obligate carrier dams were heterozygous. From the 403 animals that were sampled in 2016 and 2017, 28 animals were heterozygous and the remaining 375 animals were homozygous wildtype. An estimated allele frequency of 3.5% was calculated for the current herd that was sampled.

### Characterisation of NPC1 phenotype in fibroblast culture

The retrospective nature of this study has meant that since the report of the affected calves in 2005, no recent cases have been available. Therefore, only fibroblast cell cultures for calf 2 and calf 3 were available for further phenotype analysis.

**Fig 5:**
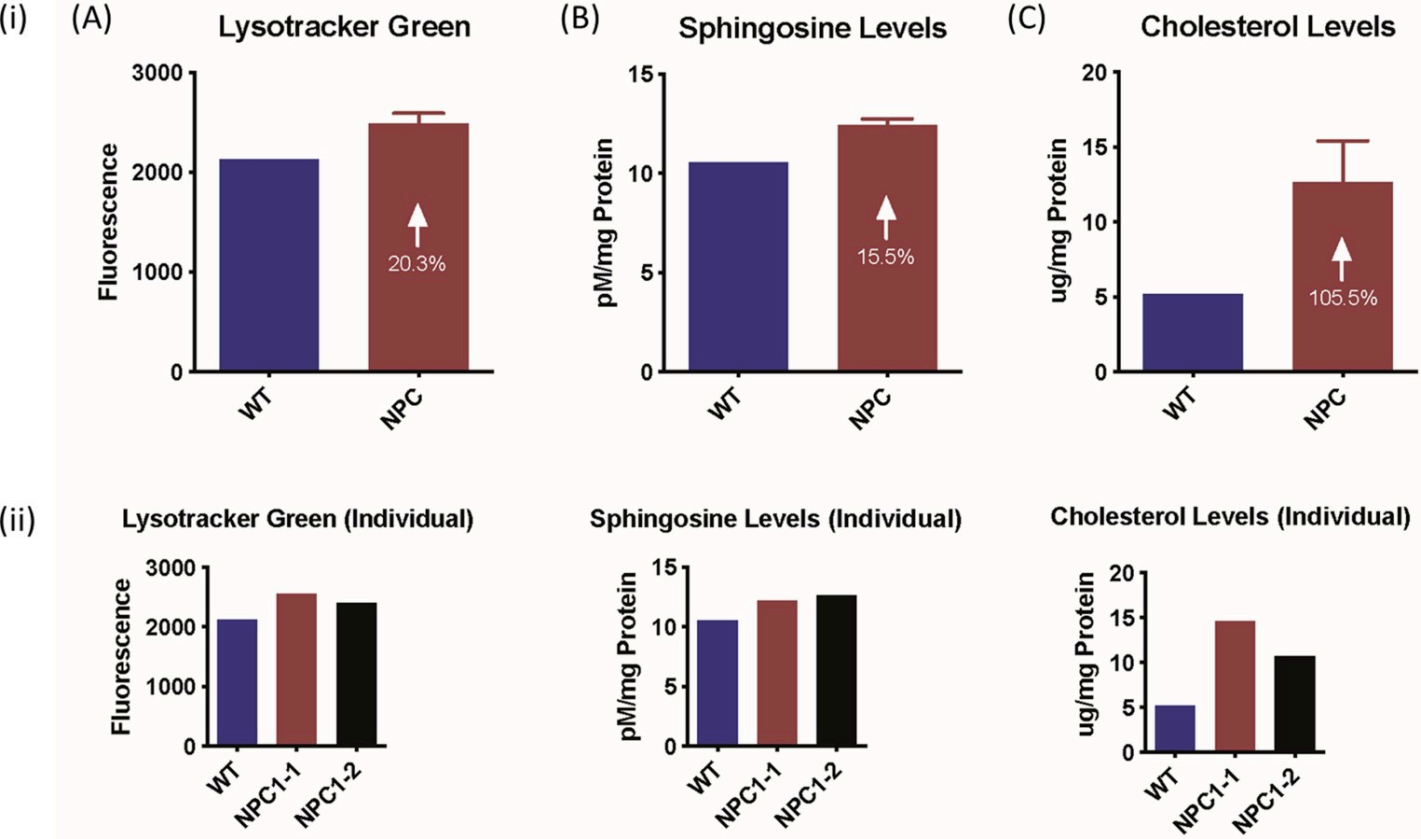
Characterisation of NPC1-mutant bovine fibroblasts for (i) wildtype (WT) and NPC1-affected calves (NPC), consisting of calves 2 and 3 and (ii) individual profiles. (A) Total acidic compartment volume measurements with Lysotracker Green fluorescence values (mean, standard deviation (SD)). (B) Total sphingosine levels with samples labeled with o-phthaldialdehyde solution (mean, SD). (C) Total cholesterol levels measured with Amplex Red cholesterol assay, followed by Folch extraction (mean, SD).

**Fig 6:**
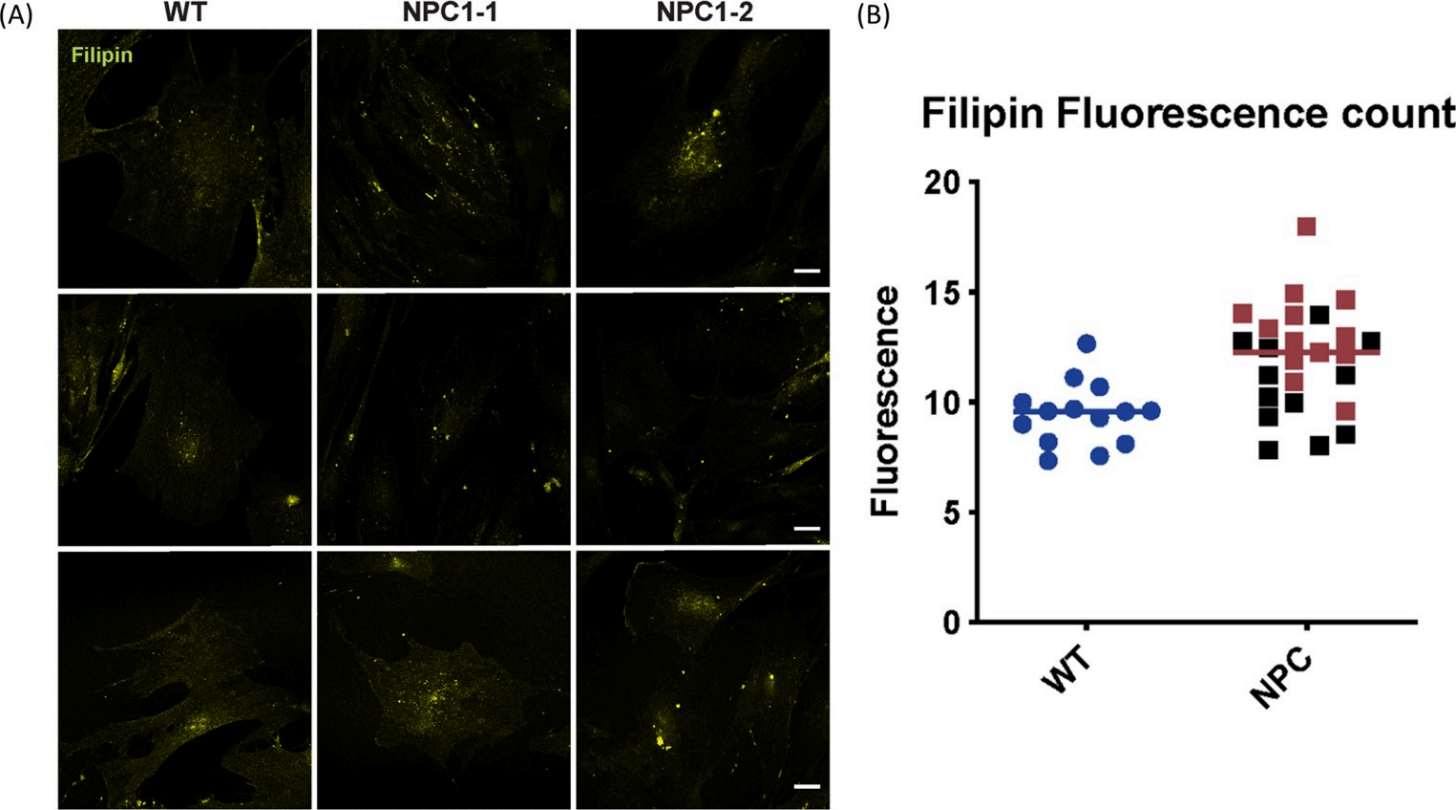
Filipin staining for free cholesterol in cells in wildtype Angus fibroblast cells (WT) and affected calves 2 (NPC1-1) and 3 (NPC1-2), scale bar is equal to 30 microns. (A) Representative images for filipin staining (yellow) for wildtype and affected calves, with vertical tiles under each sample. (B) Fluorescence quantification of filipin staining. NPC1-1 (calf 2) is represented in red and NPC1-2 (calf 3) in black. Mean gray value was quantified for each cell with Fiji (image J) software.

**Fig 7:**
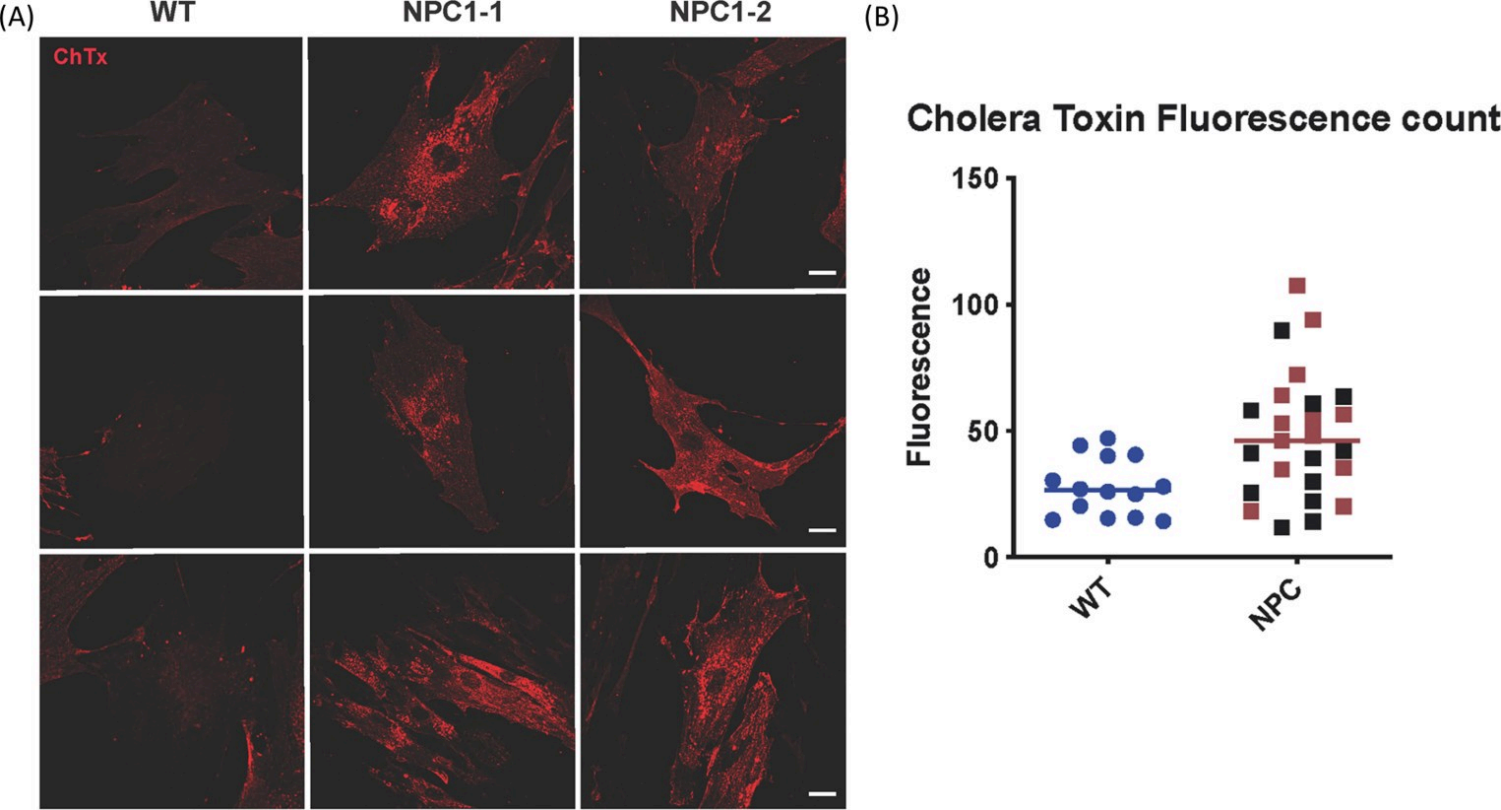
Cholera toxin B staining for GM1 localisation in cells in wildtype Angus fibroblast cells (WT) and affected calves 2 (NPC1-1) and 3 (NPC1-2). (A) Representative images for cholera toxin staining (red). Scale bar 30 micron. (B) Fluorescence quantification of cholera toxin staining. NPC1-1 (calf 2) is represented in red and NPC1-2 (calf 3) in black. Mean gray value was quantified for each cell with Fiji software.

Specific α-mannosidase enzyme activities for the control, calf 2 and calf 3 were 126, 147 and 135 nmol/hr/mg protein respectively, and did not reflect results consistent with α-mannosidosis. Characterisation of fibroblast cultures from calf 2 and calf 3 and an unrelated Angus control confirmed the NPC phenotype. In the affected animals, the overall acid compartment measured in the Lysotracker analysis, sphingosine levels and cholesterol levels were elevated by 20.3%, 15.5% and 105.5% respectively (Fig 5). The accumulation of unesterified cholesterol in the late endosomal/lysosomal compartments in the fibroblasts of affected calves was visualised by filipin staining (Fig 6). Cholera toxin B staining (Fig 7) showed the accumulation of ganglioside GM3 in perinuclear vesicular structures due to the impairment of the recycling of the GM3/Cholera toxin complex typical for NPC disease.

**Fig 8:**
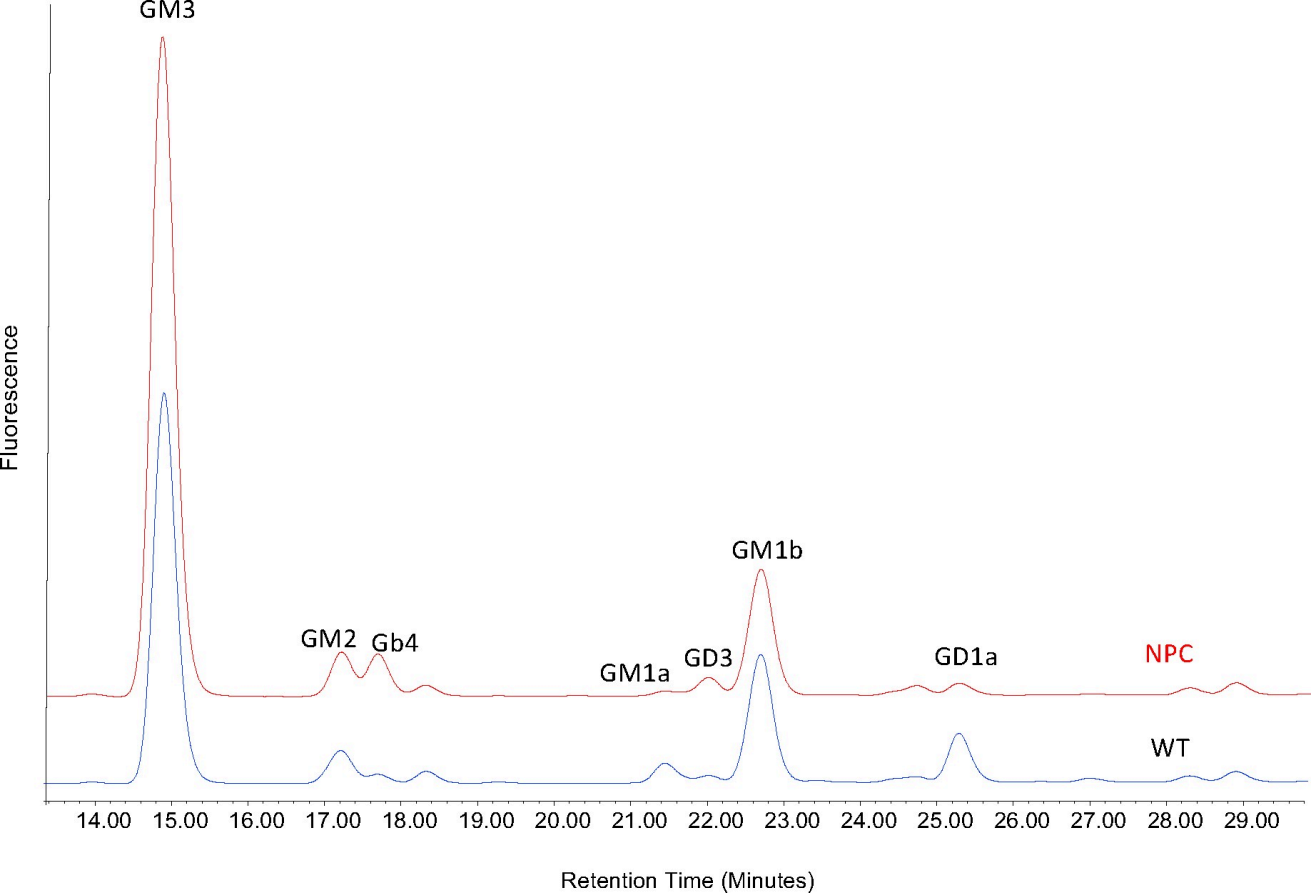
HPLC of 2AA-labelled GSL-derived glycans from bovine cultured fibroblasts. Fluorescence profiles for affected calf 2 (NPC1-1) are shown in red (NPC) and a wildtype Angus animal in blue (WT).

The HPLC of 2AA-labelled GSL-derived glycans from bovine cultured fibroblasts showed that, as observed in cultured human fibroblasts from patients with NPC, some GSLs remain constant such as GM2 and GM1b, whereas others are significantly elevated, such as GM3 or decreased, as for GM1a and GD1a, in the samples from the affected calves (Fig 8).

### Structural analysis

#### Destabilising effect of p.P990R

Both SIFT and PolyPhen-2 indicated p.P990R to be a variant that is damaging to protein function in the context of the human NPC1 protein. To further investigate the effect of this variant on protein structure though homology modelling, molecular dynamics simulations and forcefield based energy functions, such as Rosetta and FoldX were conducted. In the absence of a bovine NPC1 structure, a homology model based on a recent human NPC1 cryoEM structure (PDB: 6UOX), was built. As described in the methods section, this model was built for the wildtype protein before introducing the p.P990R mutation (Fig 10A). Comparative analysis of the wildtype and R990 models showed no steric clashes on mutagenesis, however, the charged nature of arginine may interfere with or weaken the extant salt bridge between R980 and D994 (Fig 10B). To better understand the effect of the p.P990R mutation over time, we embedded the homology models into a 1,2-palmitoyl-oleoyl-sn-glycero-3-phosphocholine (POPC) membrane and performed 500 ns of molecular dynamics (MD) simulation of the wildtype and p.P990R mutant protein in triplicate.

The MD simulations showed a small overall increase in root mean square deviation (RMSD) of the R990 variant in comparison to wildtype (mean RMSD of 7.3 Å vs. 6.5 Å over Cα atoms) (Fig 10C). Although this is not a substantial difference, further examination of the simulation trajectories revealed the spatial rearrangement and distortion of the C-terminal domain (CTD) and N-terminal domain (NTD) in R990, which are absent in the wildtype protein (Fig 10D and S2 Video). Specifically, the simulations show that the introduction of R990 weakens the extant salt bridge of R980 and D994, presumably by competing with R980. This results in larger distances between R980 and D994 over the lifetime of the simulation (Fig 10E). By weakening this salt bridge, we observed a larger degree of dynamic motion in the CTD of the R990 mutation when compared to wildtype, and this appears to have a knock on effect within the NTD, which also exhibits a large degree of motion and structural distortion (Fig 10D and 10F and S2 Video).

## Discussion

Here, we present the first report detailing the clinical signs, pathology, biochemistry and genetic characterisation of Niemann-Pick type C (NPC) disease in three Australian Angus/Angus-cross calves.

After review of case history and results from initial clinical investigations, a genetic cause of disease was proposed. A lysosomal storage disease (LSD), α-mannosidosis, which was first reported in Australian Angus cattle in 1957 [61] was considered. Cattle diagnosed with α-mannosidosis display head tremors, ataxia, incoordination and failure to thrive, leading to premature death [61]. Affected calves in this study presented with neurological signs, but in contrast to animals affected with α-mannosidosis, calves in this study were reported to have good body condition at onset of clinical signs. Genotyping of the two known bovine α-mannosidosis mutations [36, 37] in calf 1 showed that the calf was homozygous wildtype for both loci. The α-mannosidase assay showed that specific activity in fibroblasts from the two affected calves was similar to that of the control. These results, when combined with the homozygous wildtype genotype of calf 1 for both Angus and Galloway bovine α-mannosidosis mutations, excluded α-mannosidosis.

Plant toxicity or viral infection were also considered as potential causes of disease. Ingestion of legumes from the *Swainsona* spp. by livestock is known to cause clinical signs and pathological manifestations similar to those observed in inherited LSDs [62]. More specifically, the alkaloid swainsonine found within the *Swainsona* spp. inhibits α-mannosidases, thus causing a disease that is phenotypically similar to mannosidosis [63, 64]. The report of only three affected calves from this property as well as the lack of any known toxic plants suggests that acquired LSD through plant toxicity was not the causal factor for disease. Viral infection has been demonstrated in a range of neurodegenerative diseases in livestock that result in variable abnormalities of the central nervous system (CNS) [65]. Both BVDV and the Akabane virus are known to cause congenital abnormalities, notably cerebral hypoplasia and hydranencephaly depending on the foetal stage of infection [65, 66] but were excluded in all three cases.

Histopathology revealed degenerated neurons and widespread foamy vacuolation of the cytoplasm and glia of the CNS in all three affected calves. The accumulation of storage material within the CNS and peripheral organs in the affected calves resulted in a diagnosis of a LSD. Based on the clinical investigation, pedigree information, histopathology and initial filipin staining results, NPC was considered and was later confirmed with biochemical characterisation of fibroblast cells.

Initial filipin staining of fibroblast cells in 2005 produced inconclusive results due to variability in staining across cells. However, filipin staining can result in variable staining patterns in human NPC fibroblasts, and based on the variation, staining patterns are described as either classic, intermediate or variant phenotype [67, 68]. Particular *NPC1* mutations located within the C-terminal luminal domain of the human NPC1 protein [67, 69] are often associated with the variant pattern of filipin staining.

**Fig 9:**
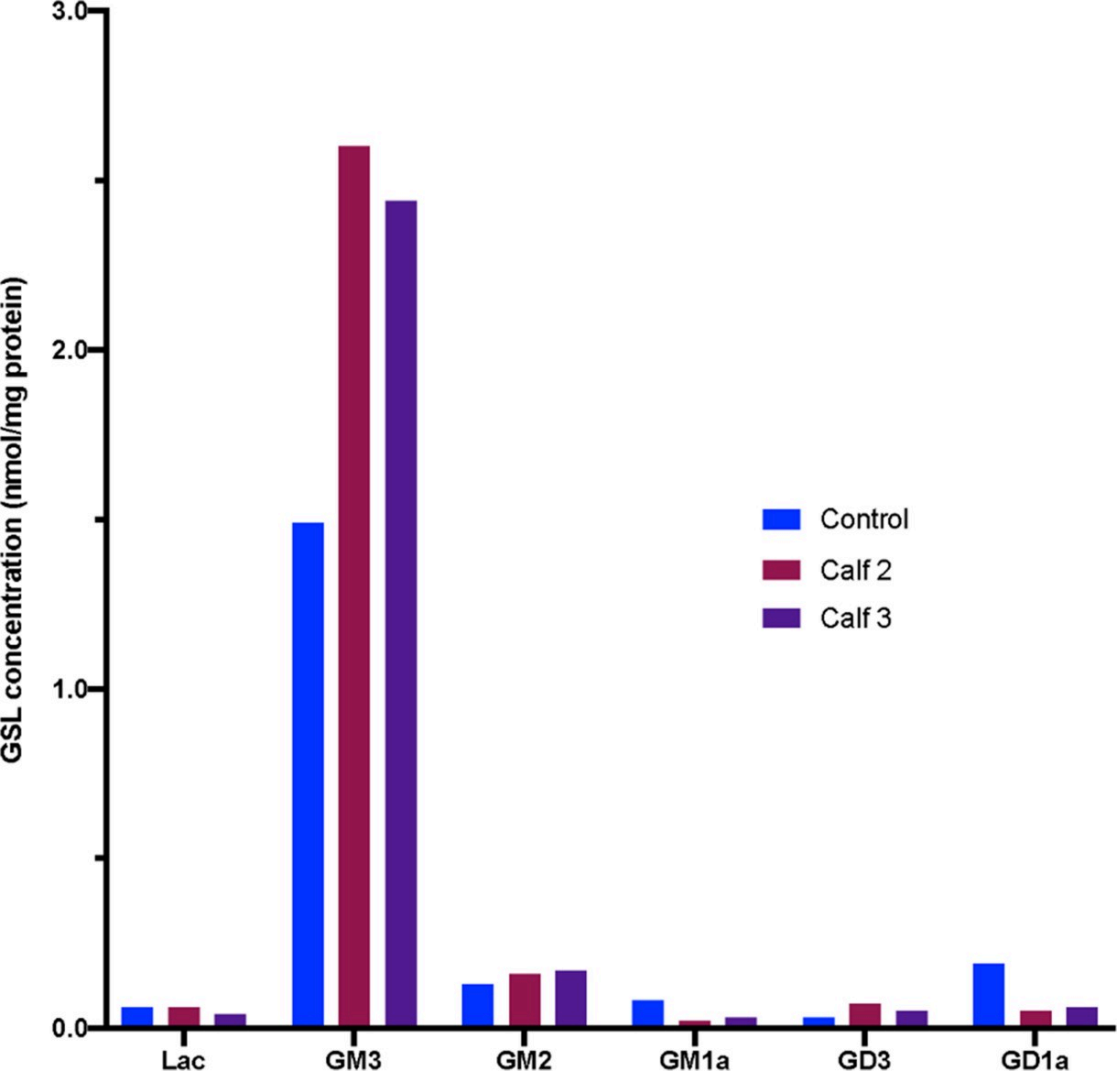
Quantification of Glycosphingolipids (GSLs) species in bovine cultured fibroblasts from HPLC analysis for wildtype (control), calf 2 (NPC1-1) and calf 3 (NPC1-2).

After identification of a possible causative mutation in *NPC1* in the affected calves, central to the diagnosis of NPC in this study was the characterisation of fibroblast cell cultures from affected calves 2 and 3 in 2017. The cells displayed elevated total acidic compartment volume measurements via Lysotracker Green analysis (Fig 5A) and increased sphingosine (Fig 5B) and total cholesterol (Fig 5C) levels. The elevation in Lysotracker staining, GSLs and cholesterol is consistent with that observed in human patients with NPC [70]. Biochemically, GSL expression in bovine cultured fibroblasts is slightly different to that in man. In control human fibroblasts, Gb3 and GM3 are the most prominent gangliosides, with small amounts of GM2 and Gb4. In human patient NPC fibroblasts, increased amounts of both GM3 and GM2 are observed (Platt lab, Oxford, unpublished data). In the bovine control fibroblasts, the most prominent GSLs are GM3 and GM1b with small amounts of GM2 and Gb4 (Figs 8 and 9). In the bovine NPC fibroblasts an almost two-fold increase in GM3 was observed, similar to those seen in human patient samples, but GM2 was unchanged. Cholera toxin can bind to a number of different gangliosides, as well as GM1, with varying affinities [71]. From the HPLC analysis (Figs 8 and 9), GM1a is less abundant in the control than in the affected calf fibroblasts. Thus, the cholera toxin binding seen in Fig 7 is likely to be a result of interaction with GM3. The total concentration of GSLs in the control bovine fibroblasts are similar to the values we have observed in human fibroblast cultures. These tend to remain very constant in healthy patient samples (Platt lab, Oxford, unpublished data). Overall the bovine biochemical data is very similar to observations made in human NPC patient fibroblasts and thus strongly suggests that these calves do exhibit phenotypes of NPC disease.

**Fig 10:**
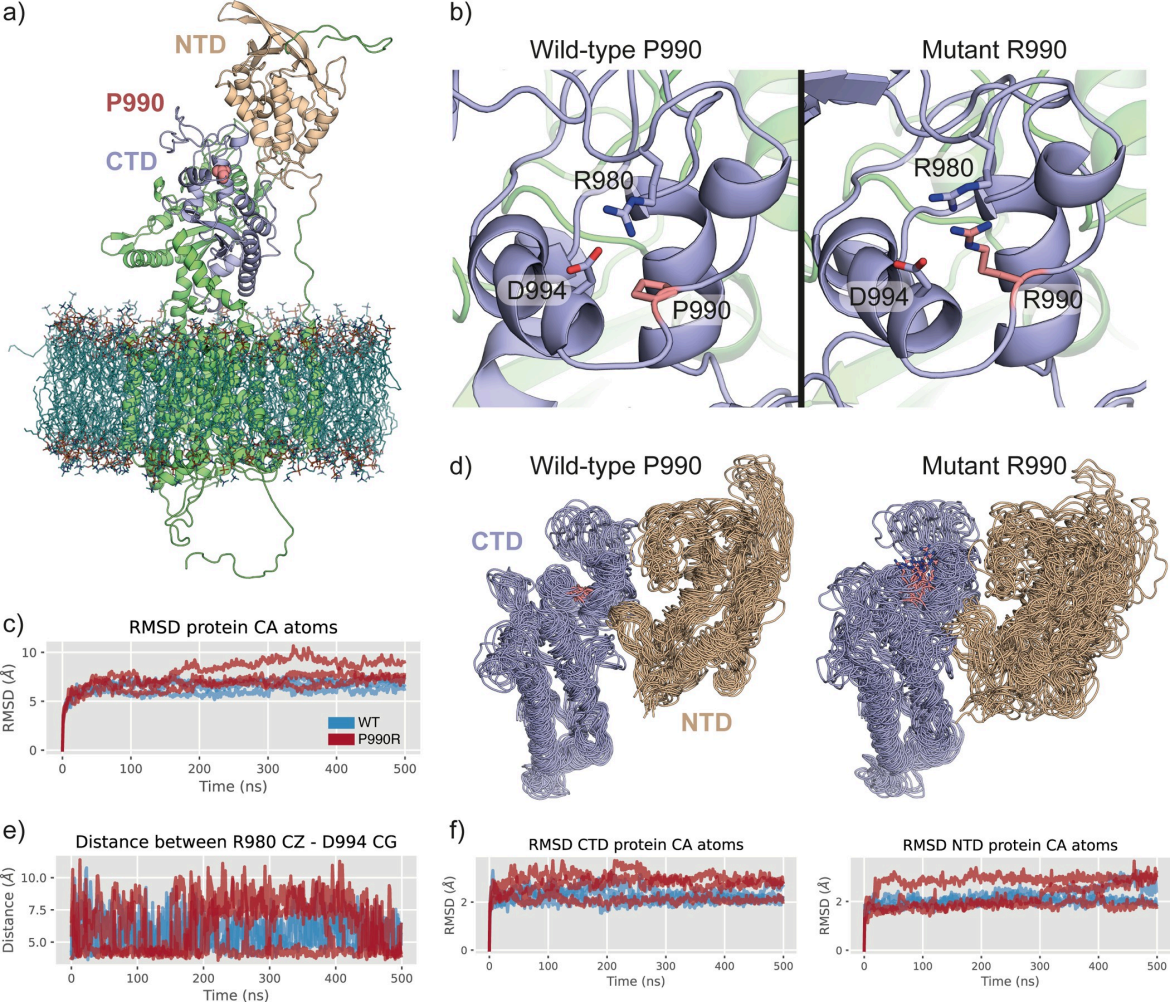
Structural modelling and simulations of bovine NPC1 wildtype and p.P990R mutant. (a) The homology model of bovine NPC1 wildtype embedded in a POPC membrane, showing the NTD in tan, the CTD in purple and the P990 position in pink. (b) Coordination of the R980-D994 salt bridge surrounding the P990 position in wildtype (left) and R990 mutant (right), showing how R990 competes for the negative charge of D994. This snapshot comes from an equilibrated state of the homology model. (c) Root mean square deviation (RMSD) plot of all protein Cα atoms for wildtype (blue) and R990 mutant (red), over 500 ns of simulation and for each replicate. (d) Superimposed simulation snapshots (every 100ns) over the simulation replicates shows structural distortions and enhanced conformational sampling of the CTD (purple) and NTD (tan) domains in the presence of R990 (pink). (e) Distance plot that measures the distance between the R980 CZ and D994 CG atoms over time for wildtype (blue) and R990 mutant (red). (f) RMSD plots showing the larger dynamic motion of the CTD (left) and NTD (right) domains in the R990 (red) mutant simulations in comparison to wildtype (blue).

To characterise the genetic basis of NPC in the affected calves, SNP genotyping and homozygosity analysis of affected calves 2 and 3 and their obligate carrier dams revealed a region of homozygosity on bovine chromosome 24 common to the affected animals only that included the location of *NPC1* (Fig 3). No region of homozygosity was observed at the location of *NPC2* on BTA10 (Fig 3). Selection of *NPC1* as a positional candidate gene was based on similar phenotype and disease progression in humans and mice, as well as gene function. Sanger sequencing of bovine cDNA revealed the homozygous missense variant NM_174758.2:c.2969C>G (NP_777183.1:p.Pro990Arg), which leads to a non-conservative amino acid change within the C-terminal luminal domain of the bovine NPC1 protein. Through SIFT analysis and PolyPhen-2 analysis, this variant is predicted to have a deleterious impact on NPC1 protein function. Multiple species alignment of the NPC1 protein (Fig 4) showed that the proline residue was conserved across all ten species. Since proline is a small non-polar amino acid and arginine is a large positively charged amino acid, this exchange may impact the structure and function of NPC1. As such, we explored homology modelling and molecular dynamics (MD) simulations of wildtype bovine NPC1 protein and the p.P990R mutant protein. Here, the homology model suggested that the p.P990R mutation might interfere with a salt-bridge (R980-D994) within the C-terminal domain (CTD) (Fig 10B). The MD simulations supported this observation, revealing an overall increase in distance between the salt-bridge (Fig 10E), which in turn appears to be responsible for increased dynamic motions of and between the CTD and NTD amongst local structural distortion of the domains (Fig 10D and 10F). Despite it being difficult to measure the effect of function in simulation, this adds extra evidence to the deleterious nature of the p.P990R mutation.

The NM_174758.2:c.2969C>G variant was observed in a homozygous state in affected calves 2 and 3 via Sanger sequencing, in a homozygous state in affected calf 1 and in a heterozygous state in the obligate carrier dams of calves 2 and 3 via genotyping. The location of NP_777183.1:p.Pro990Arg is in the C-terminal luminal domain of the NPC1 protein [72, 73]. The human NPC1 protein contains three luminal domains and 13 transmembrane domains [72]. Most causative variants within the human NPC1 protein reside within the C-terminal luminal domain spanning from residues 855 to 1098 [72, 73]. Deleterious variants within this region can result in the misfolding of the NPC1 protein, suggesting that this region has structural importance for normal protein function [11, 72, 73].

Further validation of the c.2969C>G in 403 animals was conducted using two genotyping assays. Human, feline and murine NPC is inherited via a Mendelian recessive mode of inheritance [22, 74–77]. Limited pedigree information in this study was suggestive of inbreeding and therefore a recessive mode of inheritance for the disease in these animals is proposed. Under this assumption the c.2969C>G variant segregates with the disease. Considering the location of this variant within the C-terminal luminal domain, the predicted deleterious impact of this variant on protein function and its segregation with disease, it is likely that this missense variant is responsible for the NPC phenotype observed in the Angus/Angus-cross calves.

Variants within the *NPC1* gene have been previously reported in cattle, cats and mice. Four SNPs within the *NPC1* gene were identified in Qinchuan cattle and were associated with body size traits. These variants included one missense variant and three synonymous variants [78]. The missense variant did not localise to the C-terminal luminal domain of the bovine NPC1 protein, and was instead located within loop A of the protein [78]. The cattle in that study were noted to be healthy, with no comment made in relation to any neurological or behavioural abnormalities [78]. In a colony of cats with NPC, a recessive causal variant was identified that caused similar clinical signs and biochemical profiles to human juvenile-onset NPC [22]. This variant caused an amino acid change located within the C-terminal luminal domain of the feline NPC1 protein, which is highly homologous to the human NPC1 protein [22, 79]. Three murine models of NPC have been reported, with the *Npc1*^*spm*^ and *Npc1*^*nih*^ models showing phenotypic similarity to early-onset NPC, and the recent *Npc1*^*nmf164*^ model showing phenotypic similarity to late-onset NPC in humans [74–77]. The causal variant identified in the *Npc1*^*spm*^ and *Npc1*^*nmf164*^ models similarly corresponds to amino acid changes in the C-terminal luminal loop of the NPC1 protein [77]. The *Npc1*^*nih*^ model however corresponds to an amino acid change in the second terminal loop (loop A) of the NPC1 protein, but is still associated with early-onset NPC in humans [77].

Animal models of human disease have proven useful for advancing the knowledge of the biochemical mechanisms of disease, as well as improving therapeutic studies. The feline and murine models of NPC have been used in investigating the effectiveness of treatments [80–82]. However, some doubts have been noted for the *Npc1*^*smp*^ mouse model, because it is unknown whether this model accurately reflects human NPC disease [74–76] despite the causal variant for this model being located in a region of high variant frequency [77]. While both the murine and feline animal models have contributed to human NPC research, a bovine model would be a valuable addition. Large animal models have been fundamental to discovering underlying disease mechanisms and effective therapies for lysosomal storage diseases in humans [83]. In contrast to rodent models, large animal models have a more varied genetic background, a longer lifespan, larger organs, and clinical presentation and progression of disease that are often more similar to human disease. However, breeding and maintaining research populations of large animal models is more time consuming and costly when compared to rodent models. In regard to evaluating safety and effectiveness of therapeutic interventions, large animal models are therefore recognized as important intermediates when moving from preclinical research to clinical trials in humans [83]. The development of a bovine model for human NPC disease would enable therapeutic approaches to be evaluated and implemented on a model with organ size scaling more comparable to humans than cats and rodents. Potential issues may arise when sourcing suitable reagents for testing therapeutic drug use in non-human species [84], as well as the expense of providing these therapeutic drugs to large animals over extended periods of time. Our findings of a missense variant in three Angus/Angus-cross calves represents a unique opportunity to further the knowledge of the underlying biochemistry and mechanisms of NPC in humans, especially as similar neurological clinical signs are observed between affected human patients and cattle.

For NPC, fibroblast cell cultures can be used to demonstrate the disease phenotype. Therefore, cell rescue experiments involving the overexpression of NPC1 in the established fibroblast cell line could be conducted. The cell rescue experiments would enable a reversal of the cell phenotype and thus confirm the diagnosis of NPC1. Alternatively, future work could involve the reversal of the c.2969C>G variant via CRISPR-CAS9 genome-editing [85] in affected fibroblasts to correct the phenotype.

The identification of a causative variant and a robust genotyping test to identify heterozygous individuals allowed for the effective management of this inherited diseases in the herd of origin. Genotyping of the wider Angus cattle population will reveal if the deleterious allele needs to be managed on a national or international level [86]. A bovine model for human NPC offers a unique opportunity to investigate the underlying mechanisms of the disease, as well as opportunities for targeted therapeutic approaches.

## Supporting information

S1 Fig Two genotyping assays were developed to discriminate the NM_174758.2:c.2969C>G variant for homozygous wildtype, heterozygous and homozygous mutant individuals. (a) Allelic discrimination plot visualised using QuantStudio™ Real-Time PCR System version 1.3 (Applied Biosystems™) for a TaqMan genotyping assay for homozygous wildtype (red dots), heterozygous (green dots), homozygous mutant (blue) individuals and no DNA template control (black square). (b) PCR-RFLP size discrimination visualised on a Bioanalyzer Instrument (Agilent Technologies) for (1) homozygous wildtype (230 bp, 101 bp and 44 bp) (2) heterozygous (230 bp, 186 bp, 101 bp and 44 bp), (3) homozygous mutant (186 bp, 101 bp and 44 bp) individuals and (4) no DNA template control.
(TIF)

S1 Video Video recording of an affected calf showing clinical signs including hind limb weakness, dysmetria, incoordination, walking sideways, falling over and recumbency. The video was recorded in 2005 by the producer.
(MP4)

S2 Video Simulation trajectories of wildtype (left) and p.P990R mutant (right) models. Both models were subjected to 500 ns of MD simulation and performed in triplicate (replicates 1, 2 and 3). The movie shows the NPC1 models displayed in the cartoon representation, and embedded in a POPC membrane. This movie highlights the C-terminal domain (CTD) (purple), N-terminal domain (NTD) (tan) and site of mutagenesis (pink Van der Waals spheres). The p.P990R simulations show an increased amount of motion and distortion of the CTD and NTDs internally, but also of their positions relative to each other. The full length of this movie encompasses 500 ns of MD simulation, with water molecules and ions hidden for clarity.
(MP4)
