# Correction: Molecular basis for a new bovine model of Niemann-Pick type C disease

**DOI:** 10.1371/journal.pone.0257078

**Published:** 2021-08-31

**Authors:** Shernae A. Woolley, Emily R. Tsimnadis, Cor Lenghaus, Peter J. Healy, Keith Walker, Anthony Morton, Mehar S. Khatkar, Annette Elliott, Ecem Kaya, Clarisse Hoerner, David A. Priestman, Dawn Shepherd, Frances M. Platt, Ben T. Porebski, Cali E. Willet, Brendon A. O’Rourke, Imke Tammen

The sixth author’s first name is incorrect. The correct name is Anthony Morton.
