# Supplementary figures and images for: Molecular basis for a new bovine model of Niemann-Pick type C disease
Source: PLoS One. 2020 Sep 24;15(9):e0238697. doi: 10.1371/journal.pone.0238697 (PMC7514041; doi:10.1371/journal.pone.0238697)

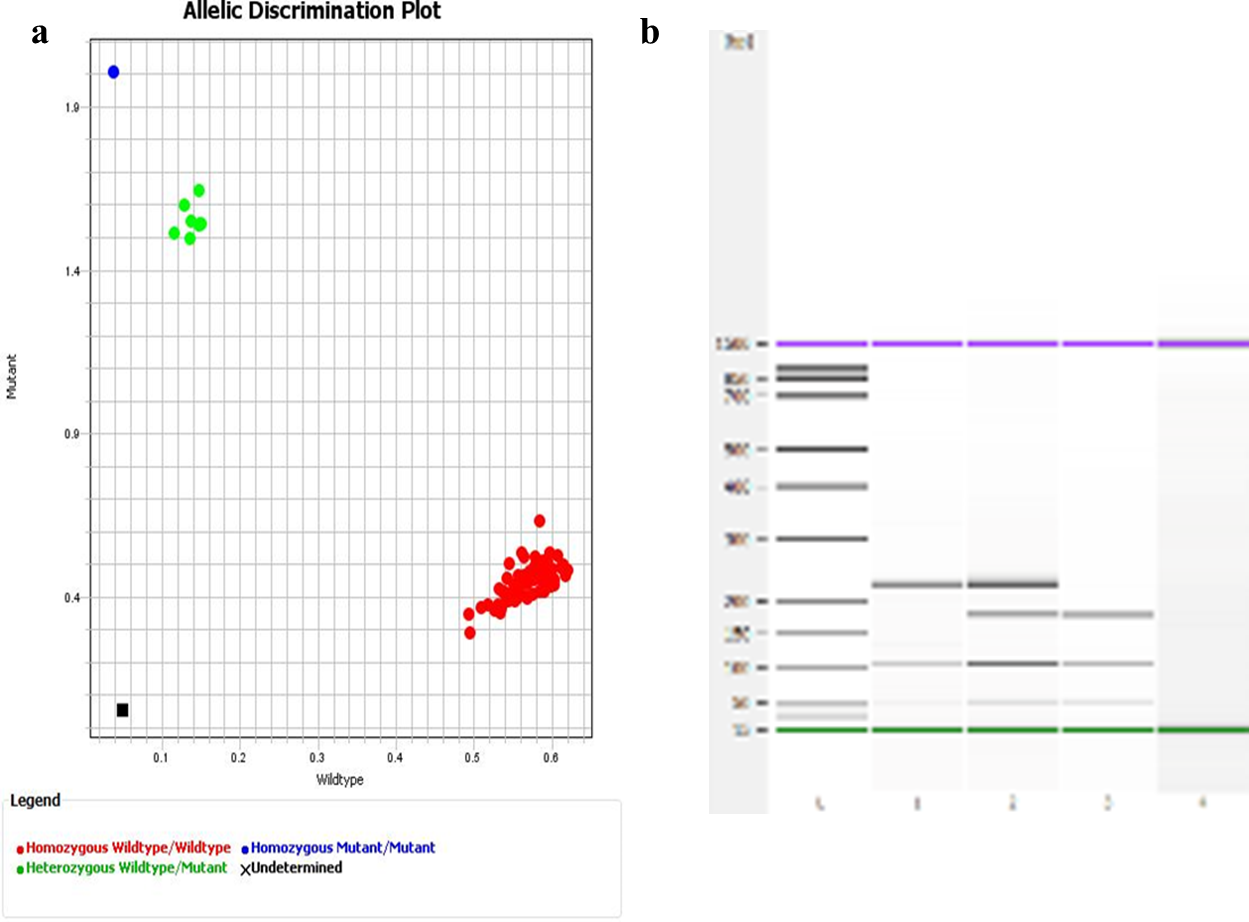

Supplement: S1 Fig — (a) Allelic discrimination plot visualised using QuantStudio™ Real-Time PCR System version 1.3 (Applied Biosystems™) for a TaqMan genotyping assay for homozygous wildtype (red dots), heterozygous (green dots), homozygous mutant (blue) individuals and no DNA template control (black square). (b) PCR-RFLP size discrimination visualised on a Bioanalyzer Instrument (Agilent Technologies) for (1) homozygous wildtype (230 bp, 101 bp and 44 bp) (2) heterozygous (230 bp, 186 bp, 101 bp and 44 bp), (3) homozygous mutant (186 bp, 101 bp and 44 bp) individuals and (4) no DNA template control. (TIF) [file pone.0238697.s001.tif]
